# Glycyrrhizin Inhibits PEDV Infection and Proinflammatory Cytokine Secretion via the HMGB1/TLR4-MAPK p38 Pathway

**DOI:** 10.3390/ijms21082961

**Published:** 2020-04-23

**Authors:** Ruyi Gao, Yongshuai Zhang, Yuhui Kang, Weiyin Xu, Luyao Jiang, Tingting Guo, Changchao Huan

**Affiliations:** 1 Institutes of Agricultural Science and Technology Development, College of Veterinary Medicine, Yangzhou University, Yangzhou 225009, China; d160095@yzu.edu.cn (R.G.); MZ120170967@yzu.edu.cn (Y.Z.); 161601112@yzu.edu.cn (Y.K.); MX120180721@yzu.edu.cn (W.X.); MZ160790@yzu.edu.cn (L.J.); 2 Jiangsu Co-Innovation Center for Prevention and Control of Important Animal Infectious Diseases and Zoonoses, Yangzhou 225009, China; 3 Key Laboratory of Avian Bioproduct Development, Ministry of Agriculture and Rural Affairs, Yangzhou 225009, China; 4 College of Medicine, Yangzhou University, Yangzhou 225009, China; ttguo1989@yzu.edu.cn

**Keywords:** glycyrrhizin, PEDV infection, proinflammatory cytokine, HMGB1, TLR4, MAPK p38

## Abstract

Our previous study showed that glycyrrhizin (GLY) inhibited porcine epidemic diarrhea virus (PEDV) infection, but the mechanisms of GLY anti-PEDV action remain unclear. In this study, we focused on the anti-PEDV and anti-proinflammatory cytokine secretion mechanisms of GLY. We found that PEDV infection had no effect on toll-like receptor 4 (TLR4) protein and mRNA levels, but that TLR4 regulated PEDV infection and the mRNA levels of proinflammatory cytokines. In addition, we demonstrated that TLR4 regulated p38 phosphorylation but not extracellular regulated protein kinases1/2 (Erk1/2) and c-Jun N-terminal kinases (JNK) phosphorylation, and that GLY inhibited p38 phosphorylation but not Erk1/2 and JNK phosphorylation. Therefore, we further explored the relationship between high mobility group box-1 (HMGB1) and p38. We demonstrated that inhibition of HMGB1 using an antibody, mutation, or knockdown decreased p38 phosphorylation. Thus, HMGB1 participated in activation of p38 through TLR4. Collectively, our data indicated that GLY inhibited PEDV infection and decreased proinflammatory cytokine secretion via the HMGB1/TLR4-mitogen-activated protein kinase (MAPK) p38 pathway.

## 1. Introduction

Porcine epidemic diarrhea (PED) is characterized by vomiting, dehydration, and watery diarrhea. The causative agent of PED is PED virus (PEDV), which emerged in Asia, America, and Europe and causes high mortality rates in suckling piglets [1,2,3]. PED was initially reported in Belgium and the United Kingdom in the late 1970s. Recently, outbreaks in swine-raising countries worldwide have led to tremendous economic losses [1,3,4,5,6,7]. PED is a significant public health concern because PEDV is a potential threat to humans [8]. PEDV, an enveloped RNA virus with a single-stranded, positive-sense genome, rapidly becomes epidemic in nearby pig farms [9,10,11].

Glycyrrhizin (GLY) is a well-characterized component of Gancao and has been used as traditional medicine. GLY is extracted from licorice, which has many pharmacological and biological activities, such as anti-tumor, anti-inflammatory, anti-oxidative, and anti-viral effects [12,13,14,15]. GLY contains one molecule of glycyrretinic acid and two molecules of glucuronic acid (823 Da) [16,17]. GLY has inhibitory effects on hepatitis C virus (HCV) [18,19], herpes simplex type 1 [20], influenza virus [21], and Kaposi’s sarcoma-associated herpes virus [22]. GLY is also a high mobility group box-1 (HMGB1) inhibitor. Extracellular HMGB1 involves a damage-associated molecular pattern to activate proinflammatory signaling pathways through toll-like receptor 4 (TLR4) [23,24]. HMGB1 is a unique mediator of inflammation-associated events and innate immune responses [25]. We previously showed that GLY inhibited PEDV [26], but the anti-PEDV mechanisms of GLY remain unclear.

The mitogen-activated protein kinase (MAPK) cascade pathways are activated by viral infection. The MAPK pathways play a critical role in transmitting signals to the intracellular environment and exquisitely controlling numerous cellular activities. Three distinct MAPKs have been identified on the basis of the respective terminal MAPK components, namely, extracellular signal-regulated kinases (ERK), MAPK p38, and c-Jun N-terminal kinases (JNK) [27,28,29]. The MAPK pathways play vital role in viral infection including foot-and-mouth disease and influenza [30,31]. Severe acute respiratory syndrome coronavirus(SARS-CoV) 3b protein induces activator protein-1 (AP-1) transcriptional activity through activation of JNK and ERK pathways, and SARS-CoV 3b protein has an important role in the stimulation of AP-1-dependent genes, especially proinflammatory cytokines causing the cytokine storm during SARS virus infection [32]. The MAPK p38 pathway is a key regulator of inflammatory responses [33,34,35,36]. Human coronavirus 229E and transmissible gastroenteritis virus wuhan 1 (TGEV WH-1) replication requires MAPK p38 activation [37,38]. Lipopolysaccharide (LPS)-induced inflammation depends on p38 MAPK signaling [39,40]. Infection and inflammation of influenza A virus are inhibited on the basis of TLR4-MAPK p38 pathways [41]. HMGB1 promotes the synthesis of proinflammatory interleukin (pro-IL)-1β and proinflammatory interleukin (pro-IL)-18 by activation of MAPK p38 [42], and acts in synergy with LPS to augment IL-6 production, which depends on MAPK p38 and nuclear transcription factor-kappa B (NF-κB) activation [43].

In the present study, we explored the anti-PEDV mechanisms of GLY. We demonstrated that the TLR4 inhibitor, TAK-242 (TAK), and mitogen-activated protein kinase p38 (MAPK p38) inhibitor, SB202190 (SB), both inhibited PEDV infection. In addition, we confirmed that HMGB1 and TLR4 functioned upstream of MAPK p38, but that this was not the case for extracellular regulated protein kinases1/2 (Erk1/2) and c-Jun N-terminal kinase (JNK). The effects of GLY against PEDV infection depended on the HMGB1/TLR4-MAPK p38 pathway.

## 2. Results

### 2.1. Effect of PEDV Infection on TLR4 in Vero Cells

**Figure 1:**
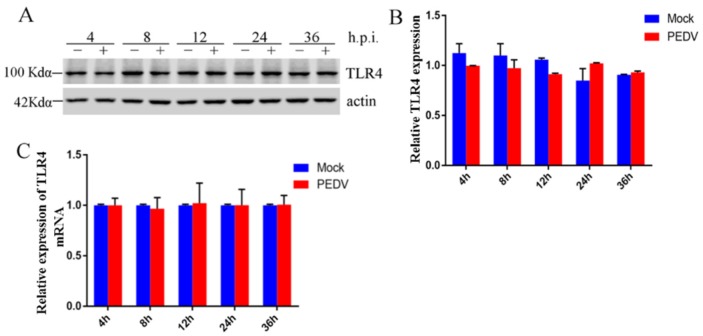
Porcine epidemic diarrhea virus (PEDV) had no effect on expression of toll-like receptor 4 (TLR4). Vero cells were infected with PEDV (0.1 multiplicity of infection (MOI)) for different lengths of time (4, 8, 12, 24, or 36 h). (**A**) Levels of TLR4 protein were analyzed by Western blotting. (**B**) Fold changes in the TLR4/actin ratio were plotted using ImageJ. (**C**) Levels of TLR4 mRNA were analyzed by qRT-PCR. Bars represent standard deviations.

GLY is a competitive inhibitor of HMGB1 cytokine activity. Our previous study demonstrated that GLY inhibited PEDV infection, and that extracellular HMGB1 binding to TLR4 promoted inflammatory responses [44]. However, there have been no studies of the effects of PEDV on TLR4. Thus, we explored the effect of PEDV infection on TLR4. Vero cells were infected with PEDV (0.1 multiplicity of infection (MOI)) for different lengths of time (4, 8, 12, 24, and 36 h). The Vero cells were collected for Western blotting and qRT-PCR. Western blotting showed no change in TLR4 protein levels following infection of Vero cells with PEDV (Figure 1A,B). In addition, qRT-PCR showed that PEDV infection had no effect on TLR4 mRNA levels (Figure 1C). These results revealed that PEDV infection in Vero cells had no effect on TLR4 activation.

### 2.2. TLR4 Regulated PEDV Infection

**Figure 2:**
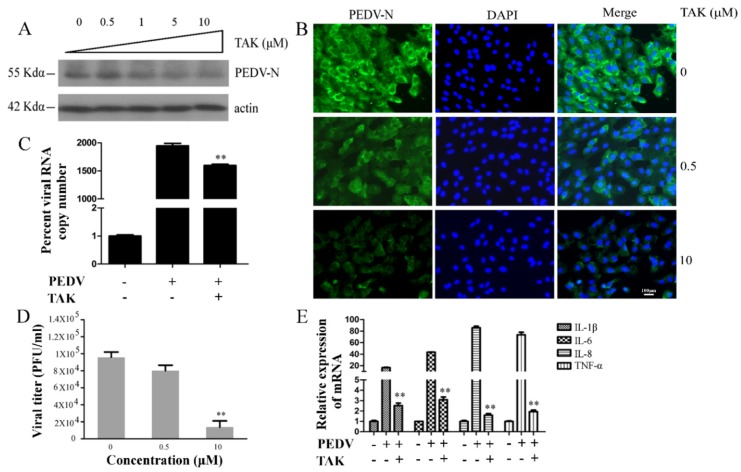
TLR4 inhibitor TAK-242 (TAK) inhibited PEDV infection and increased levels of proinflammatory cytokines. Vero cells were treated with different concentrations of TAK for 2 h and then infected with PEDV (0.1 MOI) in the presence of different concentrations of TAK for 24 h. (**A**) porcine epidemic diarrhea virus nucleocapsid (PEDV-N) expression was analyzed by Western blotting. Actin was used as a loading control. (**B**) Immunofluorescence of PEDV-N (green) detected in infected Vero cells (blue is 4’,6-diamidino-2-phenylindole (DAPI)). (**C**) Levels of PEDV open reading frame (ORF3) RNA in infected cells were determined by qRT-PCR. (**D**) Viral titers in supernatants after TAK treatment were measured using a plaque formation assay. (**E**) Levels of mRNAs encoding proinflammatory cytokines were analyzed by qRT-PCR. *p*-values less than 0.05 were considered statistically significant (** *p* < 0.01). Bars represent standard deviations.

Antiviral responses are associated with TLR4, and viral RNA and DNA can interact with TLR4. Previous studies showed that TLR4 can interact with the lipoprotein (NS1) of Dengue virus and with NS5A of HCV [3,18]. PEDV infection had no effect on TLR4 expression, but we further explored whether TLR4 regulated PEDV infection. We pretreated Vero cells with the TLR4 inhibitor TAK for 2 h and then infected the cells with PEDV (0.1 MOI) in the presence of various concentrations of TAK for 24 h. Vero cells and supernatants were collected for Western blotting, plaque formation assays, and qRT-PCR. Western blotting showed that TAK induced moderately decreased porcine epidemic diarrhea virus nucleocapsid (PEDV-N) protein expression in a dose-dependent manner, and about 73% PEDV-N protein decreased when treating with 10 μM concentration of TAK (Figure 2A), demonstrating the fact that TLR4 might regulate PEDV infection. Immunofluorescence assays (IFAs) confirmed that TAK reduced PEDV infection rate about 79% at 10 μM concentration (Figure 2B). In addition, TAK caused reductions in viral open reading frame (ORF3) mRNA levels at a concentration of 1 μM (Figure 2C). The plaque formation assay showed viral titer in the supernatant of cells was reduced by about 95% at 10 μM concentration (Figure 2D). These results suggested that TAK inhibited PEDV infection in a dose-dependent manner, and furthermore that TLR4 potentially regulated this infection. In addition, TAK reduced levels of proinflammatory cytokine mRNAs, namely, IL-1β (88%), IL-6 (93%), IL-8 (98%), and tumor necrosis factor- a (TNF-a) (97%) during PEDV infection (Figure 2E). However, TAK did not have cytotoxic effects in Vero cells at concentrations up to 10 μM after 24 h [45].

### 2.3. Effect of PEDV Infection on MAPK p38, Erk1/2, and JNK

**Figure 3:**
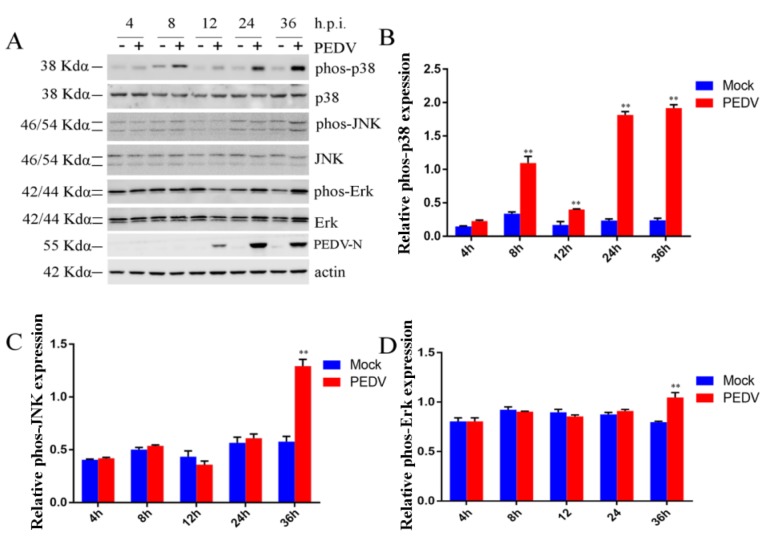
PEDV infection affected the activation of mitogen-activated protein kinase (MAPK) p38, extracellular regulated protein kinases1/2 (ERK1/2), and c-Jun N-terminal kinases (JNK). Vero cells were infected with PEDV (0.1 MOI) at 4, 8, 12, 24, and 36 h post-infection (h.p.i.). The cells were collected after different lengths of time for Western blotting. An equal amount of protein was subjected to Western blotting analysis. (**A**) Levels of phosphorylated and total MAPK p38, ERK1/2, or JNK were analyzed by Western blotting. Beta-actin was used as a loading control. (**B**) Levels of phospho-p38/total p38 were plotted using ImageJ. (**C**) Levels of phospho-JNK/total JNK were plotted using ImageJ. (**D**) Fold changes in the phospho-Erk/total Erk ratio were plotted using ImageJ. *p*-values less than 0.05 were considered statistically significant (** *p* < 0.01). Bars represent standard deviations.

We demonstrated the fact that PEDV infection was associated with TLR4, but we wanted to further explore which pathways depended on TLR4 during PEDV infection. As the MAPK pathways play a vital role in viral infection, such as foot-and-mouth disease virus and influenza A virus infection, we therefore assessed the roles of the MAPK p38, Erk1/2, and JNK pathways during PEDV infection. Phosphorylation of p38, Erk1/2, and JNK was assessed by Western blotting in Vero cells infected with PEDV (0.1 MOI) at 4, 8, 12, 24, and 36 h post-infection (h.p.i.). As shown in Figure 3A,B, PEDV infection stimulated robust phosphorylation of p38 at 8, 12, 24, and 36 h.p.i. These effects were especially apparent at 24 (4.4 times) and 36 (5.3 times) h.p.i. (Figure 3A,B). However, JNK and ERK1/2 phosphorylation were only increased at 36 h.p.i. compared with mock-infection Vero cells (Figure 3A,C,D). Levels of p38 phosphorylation were monitored during early PEDV infection and during persistent PEDV infection. However, JNK and Erk1/2 phosphorylation were only monitored at 36 h.p.i. In addition, we revealed that MAPK p38, JNK, and Erk1/2 phosphorylation were induced at 48 h.p.i., and that phosphorylation was higher at 48 h.p.i. than 36 h.p.i. [45]. Phosphorylation of p38 was induced at 24 h.p.i., whereas JNK and Erk1/2 phosphorylation were not induced until 24 h.p.i. This result suggested that p38 might play a vital role in PEDV infection from 24 h.p.i. onwards.

### 2.4. MAPK p38 Was Critical for PEDV Infection

**Figure 4:**
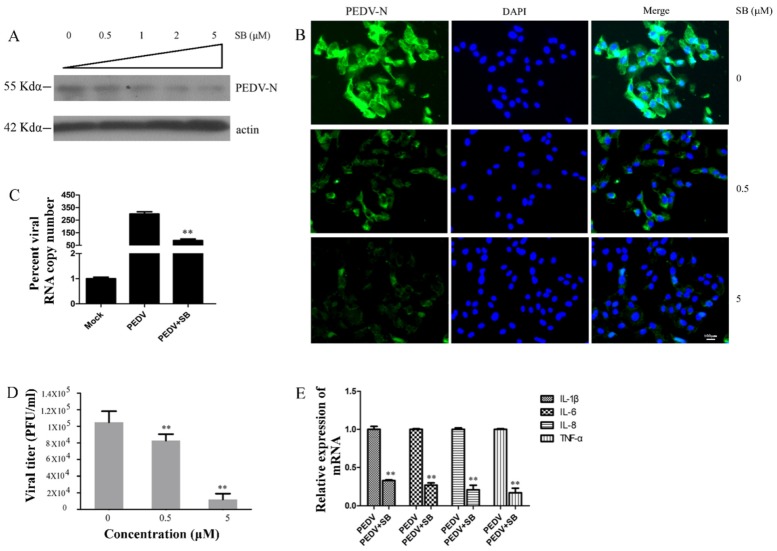
MAPK p38 inhibitor SB202190 (SB) inhibited PEDV infection and increased levels of proinflammatory cytokine production. Vero cells were treated with different concentrations of SB for 2 h and then infected with PEDV (0.1 MOI) in the presence of different concentrations of TAK for 24 h. (**A**) PEDV-N levels were analyzed by Western blotting. Beta-actin was used as a loading control. (**B**) Immunofluorescence of PEDV-N (green) detected in infected Vero cells (blue is DAPI). (**C**) Levels of PEDV ORF3 RNA in infected cells were determined by qRT-PCR. (**D**) Viral titers in supernatants after SB treatment were measured using a plaque formation assay. (**E**) Levels of mRNAs encoding proinflammatory cytokines were analyzed by qRT-PCR. *p*-values less than 0.05 were considered statistically significant (** *p* < 0.01). Bars represent standard deviations.

To explore the roles of MAPK p38 during PEDV infection, we pretreated Vero cells with different concentrations of SB for 2 h before infecting the cells with PEDV (0.1 MOI). Cells and supernatants were collected for Western blotting, plaque formation assays, and qRT-PCR 24 h after PEDV infection. We assessed the levels of PEDV-N protein by Western blotting and IFA, and found that SB inhibited PEDV-N expression in a dose-dependent manner (Figure 4A,B). Western blotting revealed that PEDV-N expression was reduced about 82% by SB at 5 μM concentration (Figure 4A), and IFA showed that PEDV infection rate was decreased about 84% by SB at the same concentration (Figure 4B). qRT-PCR showed that SB decreased the level of PEDV ORF3 mRNA about 56% at 1 μM concentration (Figure 4C). We found that PEDV titer in the supernatant was decreased about 81% at 5 μM concentration using a plaque formation assay (Figure 4D). Thus, the MAPK p38 inhibitor SB inhibited PEDV infection. In addition, levels of proinflammatory cytokine mRNA during PEDV infection were reduced about 58% (IL-1β), 61% (IL-6), 64% (IL-8), and 68% (TNF-a) by treatment with SB (Figure 4E). SB did not cause cytotoxic effects in Vero cells at concentrations up to 5 μM after 24 h [45].

### 2.5. Effects TAK on MAPK p38, JNK, and Erk during PEDV Infection

**Figure 5:**
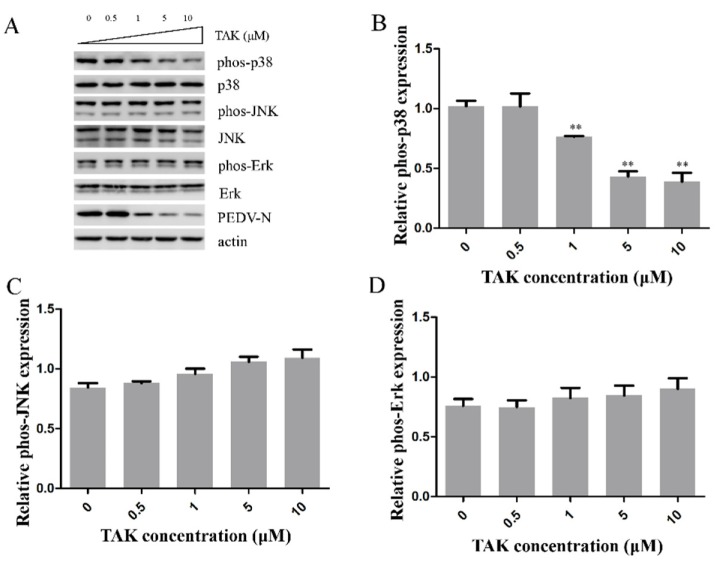
TLR4 was an upstream modulator of MAPK p38 during PEDV infection. Vero cells were treated with different concentrations of TAK for 2 h and then infected with PEDV (0.1 MOI) in the presence of different concentrations of TAK for 24 h. (**A**) Levels of phosphorylated and total MAPK p38, ERK1/2, or JNK were analyzed by Western blotting. Βeta-actin was used as a loading control. (**B**) Levels of phospho-p38/total p38 were plotted using ImageJ. (**C**) Levels of phospho-JNK/total JNK were plotted using ImageJ. (**D**) Fold changes in phospho-Erk/total Erk ratio were plotted using ImageJ. *p*-values less than 0.05 were considered statistically significant (** *p* < 0.01). Bars represent standard deviations.

Our results revealed that TLR4 and MAPK p38 regulated PEDV infection. Thus, we assessed whether TLR4 was an upstream modulator of p38 MAPK during PEDV infection. We pretreated Vero cells with TAK for 2 h and infected them with PEDV (0.1 MOI) in the presence of different concentrations of TAK for 24 h. The cells were collected to assess levels of p38, JNK, and Erk1/2 phosphorylation. TAK reduced the levels of p38 phosphorylation about 59% at 10 μM concentration during PEDV infection (Figure 5A,B), but did not inhibit JNK and ERK1/2 phosphorylation (Figure 5A,C,D). These results demonstrated TLR4 as an upstream modulator of MAPK p38 during PEDV infection, as well as the fact that PEDV infection depended on the TLR4-MAPK p38 pathway.

### 2.6. Effect of HMGB1 on MAPK p38 during PEDV Infection

**Figure 6:**
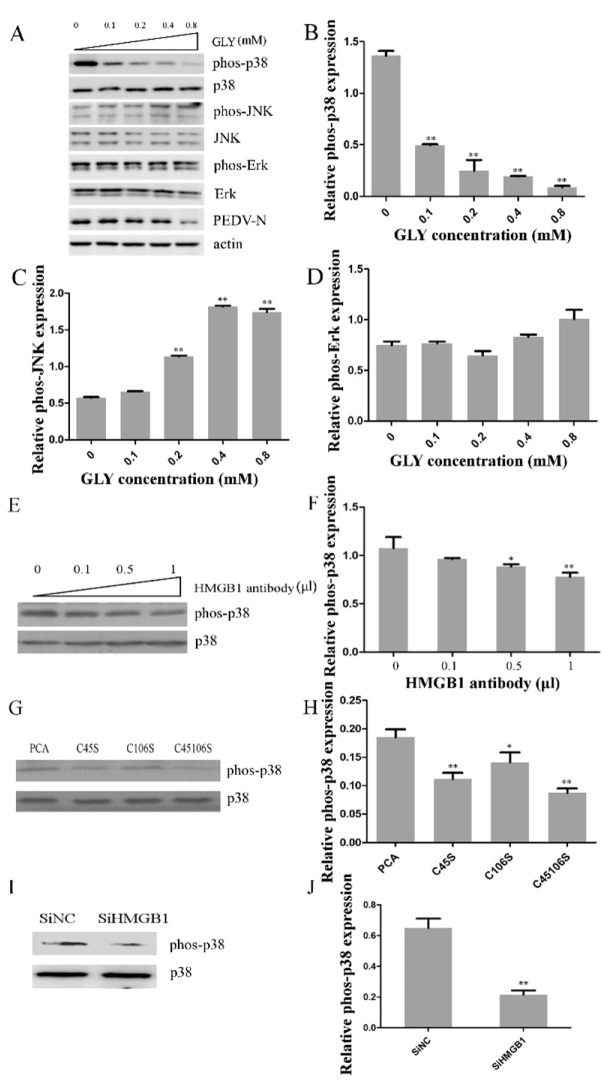
High mobility group box-1 (HMGB1) binding to TLR4 induced activation of p38 MAPK. (**A**–**D**) Vero cells were treated with different concentrations of glycyrrhizin (GLY) for 2 h and were then infected with PEDV (0.1 MOI) in the presence of different concentrations of GLY for 24 h. (**A**) Levels of phosphorylated and total MAPK p38, ERK1/2, or JNK were analyzed by Western blotting. Βeta-actin was used as a loading control. Levels of phospho-p38/total p38 (**B**), phospho-JNK/total JNK (**C**), and phospho-Erk/total Erk (**D**) were plotted using ImageJ. (**E**,**F**) Vero cells were treated with different doses of an anti-HMGB1 antibody for 2 h and then infected with PEDV (0.1 MOI) in the presence of anti-HMGB1 antibody for 24 h. (**E**) Levels of phosphorylated MAPK p38 and total MAPK p38 were analyzed by Western blotting. (**F**) Fold change in phospho-p38/total p38 ratio were plotted using ImageJ. (**G**,**H**) Vero cells were transfected with control pCAGGS (name of vector) plasmid (PCA) or plasmids encoding mutant HMGB1 (C45S, C106S, or C45S/C106S) for 24 h, and then infected with PEDV (0.1 MOI) for 24 h. (**G**) Levels of phosphorylated MAPK p38, total MAPK p38, and wild-type or mutant HMGB1 were analyzed by Western blotting. (**H**) Fold change in phospho-p38/total p38 ratio were plotted using ImageJ. (**I**,**J**) Vero cells were transfected with small interfering HMGB1 (siHMGB1) to silence HMGB1 expression for 24 h. SiNC (an irrelevant small interfering RNA (siRNA)) was used as a negative control. The cells were infected with PEDV for 24 h. (**I**) Levels of total and phosphorylated MAPK p38 and of HMGB1 were analyzed by Western blotting. (**J**) Levels of phospho-p38/total p38 were plotted using ImageJ. *p*-values less than 0.05 were considered statistically significant (* *p* < 0.05 and ** *p* < 0.01). Bars represent standard deviations.

To explore the effects of HMGB1 on MAPK p38, JNK, and Erk1/2 during PEDV infection, we pretreated Vero cells with a HMGB1 competitive inhibitor (GLY) for 2 h and infected the cells with PEDV (0.1 MOI) in the presence of different concentrations of GLY for 24 h. We collected Vero cells for Western blotting analysis. GLY inhibited p38 phosphorylation about 85% at 0.8 mM concentration induced by PEDV infection (Figure 6A,B). However, GLY did not inhibit JNK and Erk1/2 phosphorylation. These results showed that GLY inhibited PEDV infection through effects on MAPK p38. However, GLY caused little increase in JNK phosphorylation at 0.4 and 0.8 mM and little increase in Erk1/2 phosphorylation at 0.8 mM (Figure 6A,C,D). The explanations of these effects need to be explored in future studies. We performed an HMGB1 antibody neutralization experiment to investigate whether HMGB1 induced activation of MAPK p38. Vero cells were incubated with an anti-HMGB1 antibody for 24 h, and then the cells were infected with PEDV. Western blotting demonstrated that p38 phosphorylation was deceased by HMGB1 antibody treatment (Figure 6E,F). HMGB1 binding to TLR4 requires the presence of a reduced Cys106 and a disulfide bond between Cys23 and Cys45 in HMGB1 [46]. We found that mutant forms of HMGB1 (HMGB1-C45S, HMGB1-C106S, and HMGB1-C45S/C106S) decreased the activation of MAPK p38 (Figure 6G,H). In addition, small interfering RNA (siRNA) knockdown of HMGB1 inhibited the levels of p38 phosphorylation by about 66% (Figure 6I,J). Thus, HMGB1 can lead to activation of MAPK p38.

## 3. Discussion

HMGB1 is a ubiquitous and conserved DNA-binding protein that was discovered over 30 years ago and participates in DNA replication and maintenance of nucleosome structure [47,48]. Extracellular HMGB1 can activate proinflammatory signaling pathways through TLR4 [23,24]. HMGB1 is a unique mediator of innate immune responses and inflammation-associated events [25]. Our previous study indicated that PEDV infection caused release of HMGB1, and that the HMGB1 competitive inhibitor GLY inhibited PEDV infection and attenuated proinflammatory responses by inhibition of HMGB1 [44]. Therefore, we explored the relationships between HMGB1, PEDV infection, and proinflammatory cytokine production during PEDV infection. Extracellular HMGB1 can bind to TLR4. Thus, we assessed the expression of TLR4 during PEDV infection. We found that PEDV had no effect on levels of TLR4 protein or mRNA. These results were inconsistent with our hypothesis. TLR4 and MAPK p38 play an important role in influenza A virus replication and inflammation responses. TLR4 has an antiviral effect on mouse hepatitis virus [49]. The MAPK p38 pathway is a key regulator of inflammatory responses [33,34,35,36]. HMGB1 binding to TLR4 triggers cytokine release under the prerequisite of both a disulfide bond between C23 and C45 and a reduced C106 of HMGB1 [46], and our previous study corroborates the fact that extracellular HMGB1 binding to TLR4 promotes inflammatory responses [26]. Therefore, we speculated that TLR4 might regulate PEDV infection and that inflammatory responses depended on activation of signaling cascades. We explored the effect of TLR4 on PEDV infection and proinflammatory cytokine. We revealed that the TLR4 inhibitor TAK significantly attenuated viral infection, judging by the observed downregulation of viral protein, viral RNA, and viral titer, as well as decreased proinflammatory cytokine production. These results implied that although PEDV infection had no effect on TLR4, TLR4 regulated PEDV infection through activation of signaling cascades. Our previous study showed that PEDV infection caused acetylation and the release of HMGB1, which binds TLR4 [44]. The MAPK pathways are involved in cell death [50], and play a vital role in infection of coronaviruses, such as mouse SARS-CoV and hepatitis virus [51,52]. Therefore, we assessed whether TLR4 induced signaling cascades depended on the MAPK pathways in this study. We found that early PEDV infection activated p38, and that activation of p38 is continuous during PEDV infection. This phenomenon is consistent with a previous study [53]. Maximal activation of p38 was observed at 12 h.p.i. in a previous study [53], which was inconsistent with our results. In addition, activation of JNK and ERK1/2 was only observed at 36 h.p.i.; this is inconsistent with a previous study [54]. Lee et al. and Kim et al. showed that activation of JNK or ERK1/2 occurred at 12 and 24 h.p.i., but that activation of JNK or ERK1/2 was not required for productive PEDV infection at 36 and 48 h.p.i. [53,54]. The reasons underlying these discrepancies may be relate to differences between PEDV strains, and thus require further exploration. Our results showed that only p38 phosphorylation was induced at 4, 8, 12, and 24 h.p.i., suggesting that activation of p38 might have a vital role during PEDV early infection. We thus focused our efforts on p38. We explored the effect of the p38 inhibitor SB on PEDV infection and proinflammatory cytokine production. We confirmed that the p38 inhibitor SB inhibited PEDV infection and decreased proinflammatory cytokine production.

Our results revealed that TLR4 and p38 have an important role during PEDV infection. MAPK p38 is a downstream molecule of TLR4 [55,56]. However, the dependency of the effects of TLR4 on PEDV infection and expression of proinflammatory cytokines on the p38 pathway was unknown. We revealed that the TLR4 inhibitor TAK decreased phosphorylation of p38, but slightly increased levels of phospho-JNK and Erk1/2. These results revealed that TLR4 inhibition of PEDV infection and proinflammatory cytokine production depended on p38 but not JNK and Erk1/2.

**Figure 7:**
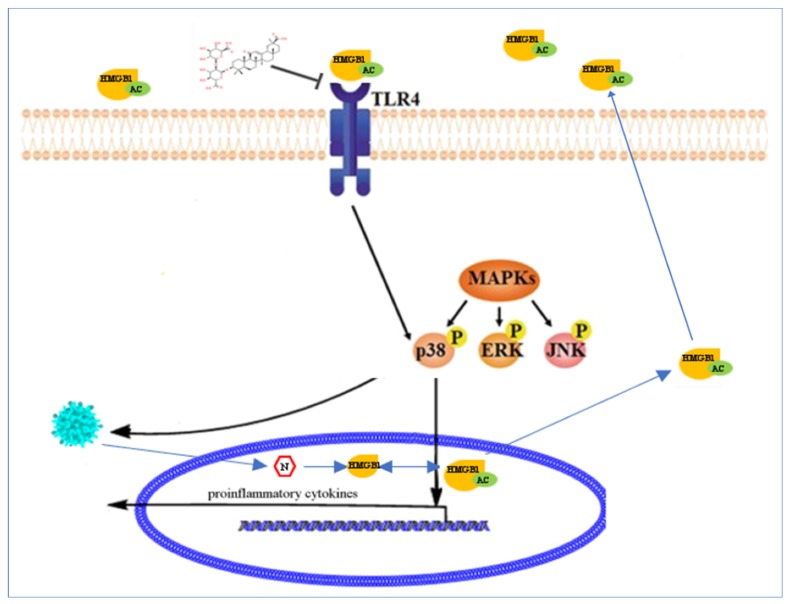
GLY prevented binding of HMGB1 to TLR4 to inhibit PEDV infection, dependent on the HMGB1/TLR4-MAPK p38 pathway.

Treatment of Vero cells infected with PEDV with GLY showed that GLY decreased the activation of p38. GLY is a competitive inhibitor of HMGB1 cytokine activity. Our previous study demonstrated that GLY could block of binding HMGB1 to TLR4 and impair the activity of TLR4 [26]. Our results showed that GLY decreased the activity of p38 dependent on TLR4 activation. However, GLY caused slight increases of phospho-JNK and Erk1/2, which was consistent with our results using the TLR4 inhibitor TAK. This phenomenon was surprising and requires further study. In addition, inhibition of HMGB1 using an antibody, site-specific mutations (HMGB1-C45S, HMGB1-C106S, and HMGB1-C45S/C106S), and siRNA knock-down decreased p38 activation. Extracellular HMGB1 can bind to TLR4, and PEDV infection leads to release of HMGB1. Our study showed that extracellular HMGB1 interaction with TLR4 leads to activation of p38, but not JNK and Erk1/2, which facilitates PEDV infection and increases proinflammatory cytokine production. GLY prevented binding HMGB1 to TLR4 to inhibit PEDV infection, and this effect depended on the HMGB1/TLR4-MAPK p38 pathway (Figure 7).

In this study, we explored GLY-inhibited PEDV infection and decreased proinflammatory cytokine production through effects on the HMGB1/TLR4-p38 pathway. Our results suggest targets for PEDV prevention and control, and provide a basis for research and development toward novel anti-PEDV agents.

## 4. Materials and Methods

### 4.1. Cells and Virus

African green monkey kidney cells (Vero cells) were cultured in high-glucose Dulbecco’s modified Eagle’s medium (DMEM; Invitrogen, Carlsbad, CA, USA) supplemented with 10% (*v*/*v*) newborn calf serum (Gibco, Carlsbad, CA, USA). PEDV (HLJBY, GenBank: KP403802.1) was propagated in Vero cells in DMEM containing 2% newborn calf serum.

### 4.2. Reagents and Antibodies

GLY and TAK were purchased from Sigma (St. Louis, MO, USA). SB was purchased from Santa Cruz. Anti-MAPK p38, phospho-p38 MAPK (Thr180/Tyr182), Erk1/2, and phospho-Erk1/2 (Thr202/Tyr204) antibodies were purchased from CST (Danvers, MA, USA). Anti-JNK and phosphor-JNK (Thr183/Tyr185) antibodies were purchased from Santa Cruz (Santa Cruz, CA, USA). The anti-PEDV-N antibody was prepared in Xiang Mao’s lab. The anti-PEDV-N polyclonal antibody was prepared in rabbits using PEDV-N protein as antigen. The work dilutions of this antiserum for Western blot and IFA were 1:5000 and 1:500, respectively.

### 4.3. Cell Viability Assay

The cytotoxic effects of TAK and SB on Vero cells were assessed using the Enhanced Cell Counting Kit-8 (CCK-8, Beyotime, Shanghai, China). Briefly, 1 × 10^4^ Vero cells per well were cultured in wells of 96-well plates containing TAK or SB. After 24 h, 10 μL of CCK-8 was added to each well, and the samples were incubated for 1 h. The absorbance of each well was measured at 450 nm. All assays were performed in triplicate.

### 4.4. Western Blotting

Western blotting analysis was performed as described previously [44], with slight modifications. Proteins in gels were transferred to a 0.22 μm nitrocellulose membrane (NC, PALL, New York, NY, USA). The membranes were blocked with 3% (*w*/*v*) nonfat milk and treated with the primary antibody hybridized with the secondary antibody, and the target protein bands were visualized via an enhanced chemiluminescence (ECL) exposure according to the producer’s instructions (Vazyme, Nanjing, China). Densitometry of TLR4, phospho-p38, phospho-Erk, and phospho-JNK expression was performed using ImageJ.

### 4.5. Immuno Fluorescence Assay (IFA)

Vero cells grown on coverslips were treated with TAK or SB and infected with PEDV at a multiplicity of infection (MOI) of 0.1 for 24 h. To assess the effect of TLR4 and MAPK p38 inhibitors on PEDV infection, Vero cells were pretreated with TAK or SB for 2 h and infected with PEDV. Twenty-four hours following PEDV infection in the presence of TAK or SB, Vero cells were fixed with 4% paraformaldehyde for 15 min and permeabilized with 0.1% Triton X-100 at room temperature for 10 min. Vero cells were blocked with 3% bovine serum albumin for 1 h and incubated with anti-PEDV-N antibody for 2 h at 37 °C The cells were washed with phosphate-buffered saline (PBS) three times and incubated with an Alexa Fluor 488-conjugated goat anti-rabbit secondary antibody (Invitrogen, Carlsbad, CA, USA) and 4′,6-diamidino-2-phenylindole (Beyotime, Shanghai, China). Fluorescence was visualized using a Leica inverted fluorescence microscope after washing three times with PBS.

### 4.6. Quantitative Real-Time PCR (qRT-PCR)

qRT-PCR was performed as described previously [53]. Total RNA was extracted from Vero cells, and gene expression was assessed by qRT-PCR using specific primers (PEDV-ORF3-F: TTTGCACTGTTTAAAGCGTCT, PEDV-ORF3-R: AGTAAAAGCAGACTAAACAAAGCCT). The samples were assessed in triplicate using SYBR (Vazyme, Nanjing, China). The data were analyzed using the 2-∆∆Ct method, and expression was normalized to that of glyceraldehyde-3-phosphate dehydrogenase (GAPDH) mRNA in the same sample.

### 4.7. Plaque Formation Assay

The plaque formation assay was carried out as previously described [53]. Briefly, Vero cells were cultured in 6-well plates containing serially-diluted virus for 1 h. Overlay medium (2% low melting-point agarose in DMEM medium containing 2% fetal bovine serum) was added to the wells for 72 h. The cells were stained with 0.5% crystal violet.

### 4.8. Statistical Analysis

All statistical data were expressed as means ± standard deviations of triplicate experiments, and the significance of differences between groups was analyzed by one-way ANOVA using the SPSS 17.0 software package (version 17.0, SPSS Inc., Chicago, IL, USA). *p*-values less than 0.05 were considered statistically significant (* *p* < 0.05 and ** *p* < 0.01).

## 5. Conclusions

In this study, we explored GLY-inhibited PEDV infection and decreased proinflammatory cytokine production through effects on the HMGB1/TLR4-p38 pathway. Our results suggest targets for PEDV prevention and control, and provide a basis for research and development toward novel anti-PEDV agents.

## Abbreviations

PEDV	porcine epidemic diarrhea virus
PED	porcine epidemic diarrhea
GLY	glycyrrhizin
HCV	hepatitis C virus
HMGB1	high mobility group box-1
TLR4	toll-like receptor 4
TAK	TAK-242
SB	SB202190
DMEM	Dulbecco’s modified Eagle’s medium
MAPK p38	mitogen-activated protein kinase p38
Erk1/2	extracellular regulated protein kinases1/2
JNK	c-Jun N-terminal kinase
CCK-8	Cell Counting Kit-8
IFA	immunofluorescence assay
